# Cognitive performance during passive heat exposure in Japanese males and tropical Asian males from Southeast Asian living in Japan

**DOI:** 10.1186/s40101-016-0124-4

**Published:** 2017-01-05

**Authors:** Titis Wijayanto, Sayo Toramoto, Yasuhiko Maeda, Su-Young Son, Sonomi Umezaki, Yutaka Tochihara

**Affiliations:** 1 Industrial Engineering Program, Faculty of Engineering, Universitas Gadjah Mada, Jl Grafika 2 Kampus UGM, Yogyakarta, 55281 Indonesia; 2 Department of Human Science, Kyushu University, 4-9-1, Shiobaru, Minami-ku, Fukuoka 815-8540 Japan; 3 National Institute of Occupational Safety and Health Japan, 6-2-1 Nagao, Tama-ku, Kawasaki 214-8585 Japan; 4 Faculty of Fukuoka Medical Technology, Teikyo University, 4-3-124 Shinkatsudachimachi, Omuta, 836-8505 Japan

**Keywords:** Cognitive performance, Passive heat exposure, Heat acclimatization

## Abstract

**Background:**

Heat acclimatization studies have reported that tropical natives have better physiological function to tolerate heat exposure compared to those from temperate natives, in which may result in a better ability to show a better resistance to performance losses during heat stress. In this study, we investigate whether the degree of heat acclimatization affects cognitive abilities during heat exposure by comparing heat acclimatization level of subjects from Southeast Asia and temperate natives from Japan.

**Methods:**

Eleven tropical males from Southeast Asia and ten temperate males from Japan participated in this study and performed two types of cognitive task: short-term memory test and mental arithmetic test, under control and passive heat exposure conditions. Passive heat condition was stimulated through leg immersion protocol by immersing subjects’ lower legs into a hot water maintained at 42 °C in a chamber controlled at 28 °C air temperature and 50% relative humidity.

**Results:**

The results show that the subjects in tropical group, who had smaller increase of rectal temperature, did not show any performance losses in both cognitive tests during heat exposure, while for Japanese group, there was performance decrement in mental arithmetic test during heat exposure (*P* < 0.05). We also found that the subjects in both tropical and Japanese groups tried to maintain their performance by increasing oxyhemoglobin in their prefrontal cortex area during performing the tasks during heat exposure. In addition, the subjects in the Japanese group showed higher increase of oxyhemoglobin when they performed the tasks during heat exposure than those when they performed the tasks in control condition (*P* < 0.05), while the subjects in tropical group did not show any differences in oxyhemoglobin during task performance between control and heating conditions.

**Conclusions:**

In addition to a better ability to maintain their homeostasis during heat exposure, tropical natives from Southeast Asia showed better resistance to performance loss during heat exposure in comparison with temperate natives from Japan. The tropical natives also showed smaller increase of oxyhemoglobin indicating less cognitive effort to maintain performance.

## Background

There have been many studies explaining physiological responses to heat exposure, but the heat exposure effects on cognitive performance are uncertain. Reviews on the heat exposure effects on cognitive performance [1–3] has been made, but it is difficult to conclude if heat exposure does [4–6] or does not [7, 8] have adverse effect on cognitive performance. Confounding factors that may affect performance during heat exposure are body temperature change brought by the exposure, dehydration level, type of task, and acclimatization level [3]. Increase in body temperature during heat exposure with some criteria of thermal conditions appears to be crucial factors affecting cognitive performance during heat exposure [1]; small changes in body temperature may have influence on human performance [1, 9, 10]. Some studies have been made to raise body temperature, and changes in cognitive performance was reported [8, 11] but failed to conclude whether heat exposure had increasing or decreasing effects. Wijayanto et al. [12] investigated the effect of passive heat exposure on cognitive performance by examining the increase of rectal temperature during passive heat exposure; they found no increasing nor decreasing effect of heat exposure on short-term memory. Along with no performance changes during heat exposure, there were greater increases of oxyhemoglobin changes (∆oxy-Hb) in prefrontal cortex (PFC) area during task performance in heat, suggesting the recruitment of available neural resources or effort by the subjects to maintain the same performance at the same level as when they felt thermally comfortable [12].

Ramsey [3] suggested that the acclimatization level of the subjects may have effects on cognitive performance during heat; acclimatized individuals who have better physiological function to tolerate heat stress should show more resistance to performance losses during heat stress compared with the non-acclimatized individuals. However, very few studies investigated acclimatization effects on cognitive performance. A study by Pepler [13] reported that young European men living in the tropics and were considered as naturally acclimatized to the tropical climate from at least 6 months to approximately 2 years showed performance deterioration in warm environment. However, residencies in tropical climate for less than 2 years may not produce significant changes in heat acclimatization level and may not have the same level of heat acclimatization as the natives; it requires at least 6 years of acclimatization to acquire the same capacity as native to that area [14]. Therefore, the subjects in Pepler’s study [13] might not have been fully acclimatized to the tropical climate and the deterioration of performance might not be due to the heat acclimatization effects. Radakovic et al. [15] reported that 10 days of heat acclimation in duration of 3 h daily, either passive or active acclimation, prevented the deterioration of attention performance during heat exposure.

Most of the studies in heat acclimatization [16–20] focused on physiological responses to heat. In terms of physiological adaptation, long-term heat-acclimatized individuals are reportedly to have smaller rises in core temperature during heat exposure [20–22] and an advantage in body fluid regulation [20], in which they indicate the ability to deal with stress from any given heat exposure [23, 24]. Although several studies have been investigating the acclimatization state of an individual during heat exposure, none of the authors specifying heat acclimatization (natural adaptation) or heat acclimation (artificial adaptation) level of their subjects in examining heat exposure effects on cognitive performance. If it is true that the degree of acclimatization or acclimation of the subjects has a role in cognitive task performance during heat exposure, therefore by examining two groups with different acclimatization or acclimation levels to hot environment should be beneficial in investigating cognitive abilities during heat exposure.

Above all, this study was conducted to investigate the effects of passive heat exposure on cognitive task performance in tropical natives from Southeast Asia, who were assumed to be acclimatized to hot environment, and temperate natives from Japan. This study sought to know whether heat adaptation level would affect cognitive task performance during heat exposure. The tropical subjects were considered to be more acclimatized to hot environment in tropical climate area and had better heat tolerance than the subjects from temperate climate, Japan. Therefore, we hypothesized that the tropical Southeast Asian subjects could retain their cognitive performance during heat exposure, as the result of long-term heat acclimatization during their life in tropical region.

## Materials and methods

### Subjects

A total of 21 male students participated as subjects in this study, 11 males from Southeast Asian countries (tropical native group (TR): four Indonesians, four Vietnamese, one Thai, one Filipino, and one Malaysian) and 10 males from Fukuoka, Japan (JP). The TR subjects were born and raised in the tropical countries that are characterized as the countries with hot and humid weather with two seasons, dry and rainy seasons. They were undergraduate and graduate students at a Japanese university and had been residing in Japan for approximately 11.9 ± 2.1 months prior to the experiments. There are no significant differences between TR and JP groups in the morphological characteristics displayed in Table 1. No major differences in dietary habits are apparent between TR and JP, and no vegetarians were recruited to participate in this study. Subjects in both groups were all students with a similar physical activity level. Most of the subjects were not currently engaged in any endurance sports activity, either personally or as a group activity. Subjects in the TR group did not report any significant change in the composition of their diet after arriving in Japan. All the subjects in both groups were all right-handed and had normal or corrected-to-normal visual acuity. Heat acclimatization status of the subjects in TR group was confirmed from the physiological responses during heat exposure in this study, that is they showed a smaller increase of rectal temperature (*T*_re_) and a small amount of total sweat rate (${\dot{M}}_{\mathrm{sw}}$) during heat exposure, that are commonly observed in tropical natives [18–23, 25, 26]. Therefore, subjects in TR group in this study could be considered as heat-acclimatized subjects. Details of the physiological responses during heat exposure in TR group are explained in the “Results” section.

**Table 1:** Morphological characteristics of tropical (TR) and Japanese subjects (JP)

	TR (*n* = 11)	JP (*n* = 10)
Age, years	25.73 ± 1.41	24.00 ± 1.03
Body height, cm	173.2 ± 1.68	170.61 ± 1.78
Body mass (W), kg	63.03 ± 1.35	60.15 ± 2.55
Body surface area (A_D_), m^2^	1.79 ± 0.03	1.73 ± 0.04
A_D_/W, m^2^ kg^−1^	0.028 ± 0.003	0.029 ± 0.003

Data are presented in mean ± SE

### Experiment protocol

**Fig. 1:**
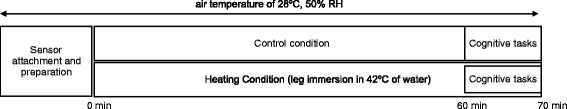
Experimental protocol for control condition (CON) and passive heating condition (HEAT)

Each subject was required to attend two sessions, control condition (CON condition), and passive heating condition (HEAT condition) as depicted in Fig. 1. Subjects wore only shorts during the experiment and maintained a sitting position on a chair in an environmental chamber maintained at an air temperature of 28 °C and 50% RH for more than 40 min to allow sensor attachment. During this period, subjects were familiarized with the cognitive tasks used in the experiment and were requested to complete a series of practice trials of the cognitive tasks to reduce learning and anxiety effects. Subjects then started CON and HEAT condition in random orders in separated days. For the CON condition, a 60-min rest in sitting position was given to the subjects before performing the cognitive tasks. In HEAT condition, a 10-min stabilization period was allowed for baseline measurement, followed by 60 min of passive heating in the same environmental chamber. Passive heating was stimulated by immersing subject’s lower legs to the knees in hot water maintained at 42 °C. Hot water leg immersion has been experimentally used previously to investigate the heating of subjects in raising body temperature and enhancing sweating [18, 19]. After 60 min of CON or HEAT conditions, subjects did two types of cognitive tasks in random order. For HEAT condition, subjects performed cognitive tasks while immersing their lower legs in hot water. Subjects underwent the second experiments at least 48 h after the first experiment. The same experimental protocol was applied to both the TR and JP subjects. The experiment was performed in winter (from the mid of January to the beginning of February) for both TR and JP groups.

### Cognitive measures

The cognitive tasks administrated in this study are Corsi block-tapping test [27, 28] assessing short-term memory and two-column digit addition test assessing mental arithmetic. Short-term memory and mental arithmetic tasks were selected as the cognitive task in this study considering the review by Pilcher et al. [29] that cognitive tasks related to memory and mathematical calculations were mostly affected by heat exposure. Both tasks were administrated using the PC version of Corsi block-tapping test and two-column digit addition operated on a free downloadable psychological test battery software, namely Psychology Experiment Building Language (PEBL) (http://pebl.sf.net) [30], projected in a monitor screen (14 in., 800 × 600 pixels) placed 70 cm in front of the subject’s eyes.

#### Corsi block-tapping test

The Corsi block-tapping test [27, 28] assesses short-term memory. In this test, subject was presented with a screen containing nine blue squares and each square lit up at 0.25 Hz in a sequence. The subject was required to remember the order in which the squares in the order they lit up. After the sequence was completed, the subject clicked on the squares in the same order with the help of a computer mouse. The first trial began with a sequence of two squares. Two trials were given per block sequence of the same length. If at least one of these trials was repeated correctly, the next two trials of a sequence of an increased length were administrated. The test was automatically discontinued when the subject failed to recall two sequences of equal length. Total score and response time were computed for each subject. The total score equals the product of Block Span and the number of correctly repeated sequences until the test was discontinued. Response time was measured from the point the sequence was completed and the subject clicked on the squares in order they lit up.

#### Two-column digit addition test

The two-column digit addition test assessed the ability to perform mathematical calculation ability. In this task, three two-digit numbers were displayed one above the other in a column in the center of the screen with a line drawn beneath. The subject’s task was to add the numbers and enter the answer using the number keys on the keypad. Once the first digit of the answer was entered, the problem disappeared. As soon as the “Enter” key was pressed, the screen was cleared and a new problem appeared. The task duration was 5 min. The numbers of correct answers and the response time were recorded as the performance indices.

### Physiological and psychological measures

*T*_re_ and skin temperatures were monitored every 2 s with thermistor probes (LT-8A; Gram Corporation, Saitama, Japan). *T*_re_ was monitored with a thermistor probe inserted 13 cm beyond the anal sphincter throughout the test, while skin temperatures were monitored at ten body sites: forehead, upper back, chest, abdomen, upper arm, forearm, hand, thigh, calf, and foot. Mean skin temperature ($\overline{T}$_sk_) was calculated using the modified Hardy and DuBois’ equation: $\overline{T}$_sk_ = 0.07*T*_forehead_ + 0.35(*T*_chest_ + *T*_abdom_ + *T*_upper back_)/3 + 0.14(*T*_upper arm_ + *T*_forearm_)/2 + 0.05*T*_hand_ + 0.19*T*_thigh_ + 0.13*T*_calf_ + 0.07*T*_foot_. Total body mass loss ($\dot{M}$_sw_) during HEAT condition and insensible body mass loss was calculated from nude body mass measured before and after each condition using a calibrated scale (METTLER ID2 MultiRange, Mettler-Toledo GmbH, August Sauter, Germany) with a minimum calibration of 1 g.

For subjective thermal measurements, each subject gave a verbal evaluation of their thermal condition using nine points rating thermal sensation (thermal sensation (TS), 4: very hot, 3: hot, 2: warm, 1: slightly warm, 0: neither, −1:slightly cool, −2:cool, −3:cold, and −4:very cold) and seven points of thermal comfort (thermal comfort (TC), 3: very comfortable, 2: comfortable, 1: slightly comfortable, 0: neither, −1: slightly uncomfortable, −2: uncomfortable, and −3:very uncomfortable).

A spatially resolved near infrared spectroscopy (NIRS) with continuous wavelength of near-infrared light at 775, 810, and 850 nm of wavelengths (NIRO-200, Hamamatsu Photonics, Japan) was used to monitor and record quantitative changes of NIRS parameters, ∆oxy-Hb, and deoxyhemoglobin change (∆deoxy-Hb), in left PFC area. We also measured The NIRS system monitors the changes in ∆oxy-Hb and ∆deoxy-Hb by using the modified Beer-Lambert law. With a path length of 24 cm, the units of oxy-Hb and deoxy-Hb are μM = 10^−6^ mol/L. The distance between NIRS optodes, emitters and detectors, was set at approximately of 4 cm between optodes [31] to allow a light penetration depth into brain tissue of approximately 1.3 cm. The optodes were housed in an optically dense black vinyl holder to ensure that the position of the optodes was fixed. The vinyl optode holder was then secured to the skin with double-sided adhesive tape and then wrapped with surgical tapes. The optodes were placed on the left side of the subject’s forehead symmetrically to measure hemodynamic responses in the left PFC with the center between emitter and detector identical to FP1 of the international 10–20 electrode system. The left PFC was measured because this part was associated to cerebral activation across cognitive tasks [32, 33]. ∆oxy-Hb and ∆deoxy-Hb were recorded at 55 to 60 min of CON and HEAT conditions. The two NIRS parameters were also measured when the subject performed the cognitive tasks.

### Data analysis

Data and figures are presented in mean ± standard error (SE). Values before subjects performing cognitive performance tasks were calculated from the last 5-min average (from 55 to 60 min) of control and passive heating condition. Change of rectal temperature (Δ*T*_re_) after 60 min of control and passive heating conditions was determined by subtracting the values during 10-min average of stabilization from the value before subjects performing cognitive tasks. ∆total-Hb was calculated from the sum of ∆oxy-Hb and ∆deoxy-Hb. PFC hemodynamics (∆oxy-Hb, ∆deoxy-Hb, and ∆total-Hb) were analyzed separately for Corsi block-tapping test and two-column digit addition test. Data were treated using two-way analysis of variance (ANOVA) for analyzing *T*_re_ *,* ${\overline{T}}_{\mathrm{sk}}$ *,* Δ*T*_re_, TC, TS, ∆oxy-Hb, and ∆deoxy-Hb, at the point of 60 min of CON and HEAT condition (group x condition). Cognitive performance results and hemodynamic changes during tasks for each group were analyzed using paired *t* test to compare CON and HEAT conditions. Percent relative changes (% changes = (HEAT-CON)/CON × 100) were calculated for cognitive task performances and then were treated using unpaired student *t* test to test group differences between TR and JP subjects. We also calculated the effect size statistics as Cohen’s *d* (standardized mean differences) to examine cognitive performance results using equation: $d=\left({\overline{X}}_{\mathrm{HEAT}}-{\overline{X}}_{\mathrm{CON}}\right)/{S}_{\mathrm{p}}$, where ${\overline{X}}_{\mathrm{HEAT}}$ is the mean performance scores of the HEAT condition, ${\overline{X}}_{\mathrm{CON}}$ is the mean performance scores of the control condition, and *S*_p_ is the standard deviation pooled across conditions. A positive *d-*score represents better performance in the HEAT condition than in the CON condition, whereas a negative *d*-score indicates worse performance. Statistical analysis was performed using SPSS for Windows version 15 software (SPSS Inc., Chicago IL, USA). *P* < 0.05 was considered as significant with *P* < 0.1 indicating a tendency toward a difference.

## Results

### Physiological and psychological strains

Table 2 depicts the physiological and psychological strains in CON and HEAT condition for TR and JP group. In comparison between TR and JP, TR group showed significantly higher *T*_re_ (*P* < 0.01) in the CON condition than those of JP group, but no differences were observed in *∆T*_re_ and insensible total body weight loss of CON condition between these two groups. TR group showed a significantly smaller Δ*T*_re_ (*P* < 0.05) and a tendency toward a lower $\dot{M}$_sw_ (*P* < 0.1) post treatment in the HEAT condition compared with the JP group.

**Table 2:** Physiological and psychophysical strains before subjects performing cognitive task after 60 min of control condition (CON) and after 60 min of passive heating condition (HEAT) in tropical group (TR) and Japanese group (JP)

	CON		HEAT	
	TR	JP	TR	JP
*T*_re_, °C	37.03 ± 0.07	36.71 ± 0.09^a^	37.49 ± 0.09^b^	37.43 ± 0.07^b^
Δ*T*_re_, °C	−0.18 ± 0.05	−0.15 ± 0.04	+0.51 ± 0.05^b^	+0.70 ± 0.06^a,b^
$\overline{T}$_sk_, °C	33.31 ± 0.15	33.34 ± 0.04	35.15 ± 0.20^b^	34.77 ± 0.19^b^
$\dot{M}$_sw_,g cm^−2^ h^−1^	20.44 ± 1.23	21.18 ± 1.74	72.42 ± 5.16^b^	90.39 ± 7.82^b,c^
TS	−0.1 ± 0.1	−0.3 ± 0.2	2.5 ± 0.3^b^	3.2 ± 0.2^a,b^
TC	0.2 ± 0.2	0.4 ± 0.2	−1.9 ± 0.2^b^	−2.2 ± 0.2^b^
Δoxy-Hb (μM)	1.32 ± 0.76	0.95 ± 0.42	14.85 ± 1.25^a,b^	18.39 ± 1.89^b^
Δdeoxy-Hb (μM)	−0.77 ± 0.27	−0.40 ± 0.15	2.02 ± 0.59	2.19 ± 0.80
Δtotal-Hb (μM)	0.55 ± 0.89	0.49 ± 0.52	16.88 ± 1.71^b^	21.43 ± 2.15

Data are presented in mean ± standard error

*T*_*re*_ rectal temperature, $\overline{T}$_*sk*_ mean skin temperature, *ΔT*_*re*_ rise of rectal temperature, $\dot{M}$_*sw*_ total sweat rate, *TS* thermal sensation, *TC* thermal comfort, *Δoxy-Hb* concentration change of oxyhemoglobin, *Δdeoxy-Hb* concentration change of deoxyhemoglobin, *Δtotal-Hb* concentration change of total hemoglobin

^a^significant difference at *P* < 0.05 between groups.

^b^significant difference at *P* < 0.05 between condition

^c^tendency toward *P* < 0.1 between groups.

All subjects from both groups reported greater thermal discomfort during HEAT condition than CON condition. Subjective reports on TS and TC showed statistical differences between HEAT and CON conditions. TS reports indicated that both TR and JP subjects felt hotter in HEAT condition than in CON condition (*P* < 0.05). Comparison between groups in the HEAT condition showed that TS of JP group was significantly higher than TR group (*P* < 0.05). TC reports showed that HEAT condition produced more thermal discomfort than CON condition (*P* < 0.05).

There were higher pre-task ∆oxy-Hb and ∆total-Hb in HEAT condition than in CON condition (*P* < 0.05), while no significant differences in ∆deoxy-Hb between HEAT and CON condition were observed for both JP and TR groups. Group comparison showed significantly higher ∆oxy-Hb in JP group compared with TR group during HEAT condition (*P* < 0.05), while no difference was observed in CON condition. There were no significant differences in ∆total-Hb and ∆deoxy-Hb between the two groups both in CON and HEAT conditions. Correlation analysis between ∆oxy-Hb and TS revealed a positive correlation between these two variables (*r* = 0.46, *P* = 0.03), that is the higher ∆oxy-Hb on prefrontal cortex, the higher thermal sensation reported by the subjects during HEAT condition.

### Cognitive task performance

#### Corsi block-tapping test performance

**Fig. 2:**
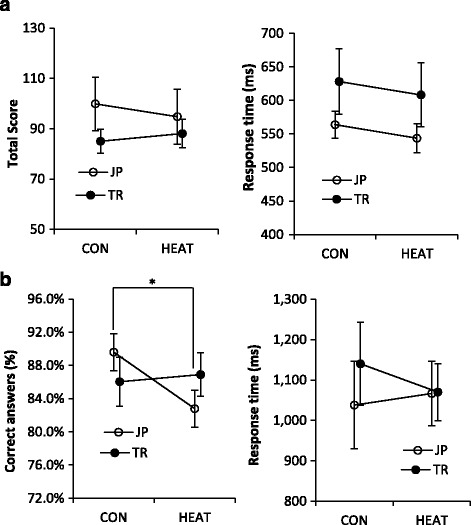
Cognitive task performance of (**a**) Corsi block-tapping test and (**b**) Two column digit addition test during control condition (CON) and passive heating condition (HEAT) in tropical (TR), Japanese (JP), and overall groups. *Asterisk* indicates significantly different at *P* < 0.05

Figure 2a depicts total score and response time of Corsi block-tapping test. Statistical analysis revealed that total score and response time did not significantly change during HEAT condition in the TR and JP groups. Small effect sizes of Corsi block-tapping test were observed for total score and response time in both TR and JP groups (Table 3). There were no significant differences in the change of total score and response time between TR and JP groups.

**Table 3:** Performance changes and effect size analysis on cognitive performance

	TR		JP	
	%Change	Effect size	%Change	Effect size
Corsi block-tapping test				
- Total score	5.2 ± 6.4	0.18	−5.3 ± 9.2	−0.16
- Response time	−0.4 ± 7.6	−0.12	−3.3 ± 3.0	−0.28
Two-column digit addition				
- Correct answer	1.3 ± 2.4	0.09	−7.2 ± 3.0*	−0.96
- Response time	−6.1 ± 6.7	−0.24	2.8 ± 9.8	0.10

*TR* tropical group, *JP* Japanese group

*significantly different between TR and JP at *P* < 0.05

#### Two-column digit addition performance

Correct answer and response time of two-column digit addition test are displayed in Fig. 2b. For TR group, there were no significant differences in correct answer and response time between CON and HEAT conditions (*P* > 0.05). In contrary, correct answer of this task performance was impaired during HEAT condition in the JP group (*P* < 0.05, Fig. 1b), although the response time was not significantly different (*P* > 0.05). A large effect size was observed for correct answer for JP group (*d* = −0.96) with a significantly greater % change of correct answer compared with the TR group (*P* < 0.05, Table 3). Meanwhile, small effect sizes were observed for correct answer in TR group (*d* = 0.09) and response time in both the TR and JP groups.

### Hemodynamic changes during cognitive task performance

#### Hemodynamic changes during Corsi block-tapping test

**Fig. 3:**
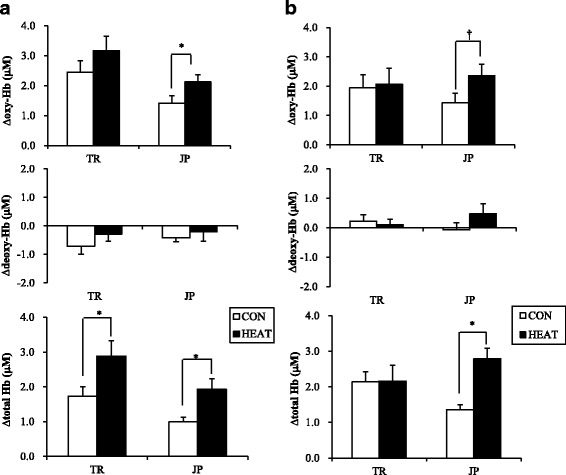
Concentration changes of oxyhemoglobin (∆oxy-Hb), concentration change of de-oxyhemoglobin changes (∆deoxy-Hb), and concentration change of total hemoglobin (∆total-Hb) during performing (**a**) Corsi block-tapping test and (**b**) Two colum digit addition test in the tropical (TR) and Japanese (JP) groups during control (CON) and passive heating (HEAT) condition. *Asterisk* indicates significantly different at *P* < 0.05. *Dagger* indicates a tendency toward *P* < 0.1.

Both group showed higher ∆oxy-Hb during Corsi block-tapping performance in HEAT condition compared with CON condition (Fig. 3a). JP group showed significantly higher ∆oxy-Hb (*P* < 0.05) when performing the task in HEAT condition compared with CON condition, while TR group did not show any significant difference in ∆oxy-Hb during task performance between HEAT and CON conditions. ∆deoxy-Hb during task performance did not differ in both conditions in JP group, either in TR group. ∆total-Hb during task performance in HEAT condition was significantly higher than in CON condition for both JP and TR groups (Fig. 3a; *P* < 0.05).

#### Hemodynamic changes during two-column digit addition test

JP group showed a tendency of higher ∆oxy-Hb when performing two-column digit addition test in HEAT condition than those in the CON condition (*P* = 0.08; Fig. 3b). ∆oxy-Hb during task performance did not show any difference between HEAT and CON conditions in TR group. No significant difference in ∆deoxy-Hb during task performance between HEAT and CON conditions in TR and JP groups. Higher ∆total-Hb during task in HEAT condition than in CON condition was only observed in JP group (*P* < 0.05), while no significant difference was observed in TR group.

#### Correlation between hemodynamic changes and task performances

**Fig. 4:**
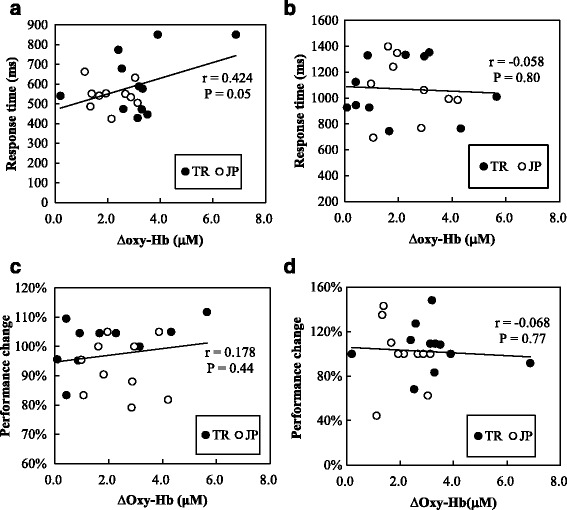
**a**, **b** Relationship between oxyhemoglobin changes (∆oxy-Hb) and response time of (**a**) Corsi block-tapping test and (**b**) two-column digit addition test. **c**, **d** Relationship between oxyhemoglobin changes (∆oxy-Hb) and performance change of (**c**) Corsi block-tapping test and (**d**) two-column digit addition test.

∆oxy-Hb during Corsi block-tapping test in HEAT condition positively correlated with the response time (*r* = 0.424, *P* = 0.05; Fig. 4a). No correlation between ∆oxy-Hb and two-column digit addition test response time was found (*r* = −0.058, *P* = 0.80; Fig. 4b). There were also no significant correlation between ∆oxy-Hb and performance changes in both Corsi block-tapping test (*r* = 0.178, *P* = 0.44; Fig. 4c) and two-column digit addition test (*r* = −0.067, *P* = 0.77; Fig. 4d).

## Discussion

Passive heat exposure induced by hot water leg immersion in this study successfully altered physiological and psychophysical strain compared with the control condition. However, heat exposure in this study has lesser effect on short-term memory during heat exposure. Both groups did not show any performance decrement during heat exposure. No alteration in short-term memory during heat stress compromised the earlier study reporting no significant effect of passive heating on short-term memory [8, 12]. The changes of rectal temperature in Japanese and tropical group were relatively small, which might indicate that heat exposure in the present study did not adequately increase the core temperature in our subjects in tropical group. Therefore, it is not surprising to observe no performance degradation in short-term memory during heat exposure in both groups, although Johnson et al. [9] and Wright et al. [10] suggested that small changes in body temperature may have influence on human performance. However, a recent review by Taylor et al. [34] suggests that core temperature alone may not be a reliable predictor of cognitive performance decline in hot environment. The task difficulty level could be the factor affecting cognitive function [1–3, 29]. In contrary to the short-term memory, no performance losses in short-term memory while mathematical abilities deteriorated during heat exposure condition in Japanese group suggested that heat exposure had different effects on different types of tasks [3, 29]. It is generally recognized that complex task performance, i.e. two-column digit addition task that requires attention and working memory, is more vulnerable than simple task performance and has been supported by recent literature reviews [1–3, 29].

Hocking et al. [11] posited the existence of cognitive reserve that can be allocated to the cognitive performance when the resources are diminished. To maintain performance level, neural resources are necessary to deal with thermal stress until the resources are overloaded [11, 35]. Tanabe et al. [36] suggests that more cerebral blood flow is necessary to maintain the same level of performance in hot environment. We observed higher increase of ∆oxy-Hb during task performance in both heat exposure and control condition for both tropical and Japanese groups during task performance. It has been demonstrated that high concentration of oxygen supply has positive effect on performance [37]. This may indicate greater increases in blood flow for prefrontal cortex activation during the task [38–40] as the result of increased cerebral perfusion and the consequence of an activated brain metabolism to support cognitive demand during the task [12, 41]. Therefore, the increase of ∆oxy-Hb during task performance in heating condition suggest the recruitment of available neural resource or effort to balance the increasing demand in cognitive performance due to external stress [11]. Additionally, there is positive correlation between ∆oxy-Hb and response time of short-term memory test. The longer the subjects allocated their extra time, the higher the ∆oxy-Hb. These findings reinforced the previous findings; the subjects tried to allocate extra time and neural resources as efforts in maintaining performance during heat stress [42]. It can be considered that subjects in both tropical and Japanese groups tried to maintain their performance by increases ∆oxy-Hb and ∆total-Hb in prefrontal cortex during performing the tasks during heat exposure.

In comparison between tropical and Japanese groups, mental arithmetic performance decreased during heat exposure in Japanese group, shown by a 7.2 ± 3.0% decrement of mental arithmetic performance (*d* = −0.96), while no performance decrement was observed in tropical group (1.3 ± 2.4% increment, *d* = 0.09). Performance deterioration in mental arithmetic task that was more vulnerable in Japanese can be explained through several reasons. First, we observed that subjects in tropical group showed a higher rectal temperature during control condition, a smaller increase of rectal temperature, and a less amount of total sweat rate during heat exposure than Japanese. A higher resting core temperature, a small increase of core temperature, and a less amount of total sweat rate during heat exposure are considered as the physiological adaptation feature of heat acclimatization reported in the previous studies [21–23, 25]. These adaptation features to tolerate heat stress become advantages for tropical subjects in this study to deal with performance decrement during heat exposure; that is, the tropical subjects in this study could maintain mental arithmetic test performance, while it was not observed in Japanese.

Second, increase of core temperature alone might not be a reliable predictor of performance deterioration [34]. Japanese subjects reported more negative feelings during heat exposure condition than tropical subjects; they felt the condition was hotter and more thermally discomfort than tropical subjects. In a study by Gaoua et al. [43], they found that subjective states of the individual may be one of the factors affecting cognitive performance and lead to alteration of complex task performance in heat. Therefore, we consider that greater thermal discomfort in Japanese may be the reason why performance decrement in mental arithmetic task was observed.

Third, ∆oxy-Hb in Japanese group were higher when they performed short-term memory and mental arithmetic task during the heat exposure compared with the control condition, while for tropical group, ∆oxy-Hb during the tasks during the heat exposure condition in the tropical group did not significantly differ to those during task performances during the control conditions. Even though higher ∆oxy-Hb and ∆total-Hb, subjects in Japanese group could not maintain their mental arithmetic task performance. Before performing the tasks during heat exposure, we observed that Japanese group showed higher ∆oxy-Hb and ∆total-Hb in heat exposure condition than tropical group. Higher ∆oxy-Hb prior to the task during heat exposure might indicate that neural resource in Japanese prior to task performance might have already be overloaded, and the arousal level might have been too high. Higher arousal level in Japanese likely led to the deterioration of task performance. It was suggested that the increase in arousal level leads to the improvement of cognitive performance, [44, 45] but it will result to performance decrement if the arousal level becomes overloaded [46].

The findings of this study support the prediction of Ramsey [3] that heat-acclimatized subjects (tropical subjects) who had a better physiological function to tolerate heat stress should be able to show more resistance to performance losses during heat stress compared to the non-acclimatized individuals (Japanese). We consider that the subjects in tropical group have been adapted to hot environment since they were born and they got used to perform any tasks that require cognitive abilities in such hot environment, which might be stressful for the opposite group. As the results of this acclimatization process, they can physiologically tolerate any given heat exposure [23, 24] and they can maintain their cognitive performance with lesser degree of cognitive efforts compared with the subjects in JP group in a thermally stressful condition.

## Conclusion

In conclusion, passive heat exposure stimulated by hot water leg immersion in this study successfully altered physiological and psychophysical strain. However, its effect on cognitive performance was more remarkable in the mental arithmetic task than in the working memory task, especially in subjects from Japan. With a better ability to maintain their homeostasis during heat exposure, the heat-acclimatized subjects from tropical Asia also showed better resistance to performance losses during heat exposure and showed lesser efforts in maintain performance compared with non-acclimatized subjects from Japan. Based on the results presented here, heat acclimatization level of an individual should be taken as consideration in examining heat exposure effects on cognitive performance.

## Abbreviations

${\overset{.}{M}}_{sw}$: Total sweat rate
${\overline{T}}_{sk}$: Mean skin temperature
${\overline{X}}_{CON}$: Mean performance during control condition
${\overline{X}}_{HEAT}$: Mean performance during heat exposure condition
∆deoxy-Hb: Deoxyhemoglobin change
∆oxy-Hb: Oxyhemoglobin change
∆total-Hb: Total hemoglobin change
CON: Control condition
*d*: Cohen’s *d* (standardized mean differences
HEAT: Heat exposure condition
JP: Japanese group
PFC: Prefrontal cortex
S_p_: The standard deviation pooled across conditions
TC: Thermal comfort
TR: Tropical native group
*T*_re_: Rectal temperature
TS: Thermal sensation
